# Assessing Dementia Needs in Illinois: A Public Health-Academic Partnership for Service Improvement

**DOI:** 10.1093/geroni/igaf122.329

**Published:** 2025-12-31

**Authors:** Julie Bobitt, Jessica Link

**Affiliations:** University of Illinois at Chicago, Chicago, Illinois, United States; Illinois Department of Public Health, Springfield, Illinois, United States

## Abstract

Alzheimer’s Disease and Related Dementias (ADRD) pose a significant public health challenge, affecting approximately 240,000 Illinois residents over age 65. Additionally, 6.1% of adults aged 45 and older in Illinois experience subjective cognitive decline (SCD), a key early indicator of dementia. Given ADRD’s progressive nature and its impact on individuals, caregivers, and healthcare systems, identifying service gaps and strengthening dementia-capable systems is critical. To address these needs, the Illinois Department of Public Health (IDPH) partnered with the University of Illinois Chicago (UIC) Center for Dissemination and Implementation Science to conduct a statewide needs assessment. This process, guided by the Needs Assessment Toolkit from the Alzheimer’s Association and the Association of State and Territorial Health Officials (ASTHO), included a landscape report, a survey of 83 professionals across aging services, healthcare, and public health, and qualitative data, collected in collaboration with state and professional organizations, from over 500 formal and informal caregivers and persons living with dementia. Findings highlight dementia prevalence and underscore the need for improved early detection, care coordination, and community-based supports. Results also provide key insights into the lived experiences of informal and formal caregivers. Challenges identified include workforce shortages, gaps in caregiver support, and the need for culturally tailored dementia care. This session will share insights from the needs assessment, highlighting efforts to enhance dementia-capable services and strengthen cross-sector partnerships. Attendees will better understand how public health-academic collaborations can drive policy and inform program development to better support persons with dementia and their caregivers.

